# Efficient design of synthetic gene circuits under cell-to-cell variability

**DOI:** 10.1186/s12859-023-05538-z

**Published:** 2023-12-07

**Authors:** Baptiste Turpin, Eline Y. Bijman, Hans-Michael Kaltenbach, Jörg Stelling

**Affiliations:** Department of Biosystems Science and Engineering (D-BSSE) and SIB Swiss Institute of Bioinformatics, ETH Zurich, 4058 Basel, Switzerland

**Keywords:** Cell-to-cell variability, Synthetic biology, Computer-aided design

## Abstract

**Background:**

Synthetic biologists use and combine diverse biological parts to build systems such as genetic circuits that perform desirable functions in, for example, biomedical or industrial applications. Computer-aided design methods have been developed to help choose appropriate network structures and biological parts for a given design objective. However, they almost always model the behavior of the network in an average cell, despite pervasive cell-to-cell variability.

**Results:**

Here, we present a computational framework and an efficient algorithm to guide the design of synthetic biological circuits while accounting for cell-to-cell variability explicitly. Our design method integrates a Non-linear Mixed-Effects (NLME) framework into a Markov Chain Monte-Carlo (MCMC) algorithm for design based on ordinary differential equation (ODE) models. The analysis of a recently developed transcriptional controller demonstrates first insights into design guidelines when trying to achieve reliable performance under cell-to-cell variability.

**Conclusion:**

We anticipate that our method not only facilitates the rational design of synthetic networks under cell-to-cell variability, but also enables novel applications by supporting design objectives that specify the desired behavior of cell populations.

**Supplementary Information:**

The online version contains supplementary material available at 10.1186/s12859-023-05538-z.

## Background

Synthetic biology aims at establishing novel functions in biological systems, or to re-engineer existing ones, in many areas such as new materials or cell-based therapies that are starting to see real-world applications [1]. The conceptual core of the field’s rational engineering approach to establish, for example, the corresponding synthetic gene circuits are a systematic design-build-test cycle and the use of predictive mathematical models throughout this cycle to design, analyze, and tune the circuits [2].

**Fig. 1:**
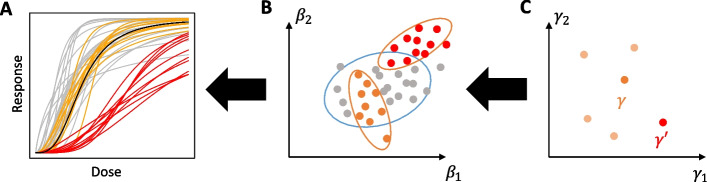
Cell behaviors relate to parameters at the individual and population level. **A** Dose-response relationships for single cells (lines) drawn from two distinct populations (red and orange) as well as other cells not belonging to any population (gray). The design objective for individual cells is represented by an ideal reference curve (black). **B** Space of individual parameters $\beta$, the set of possible parameter values for a single cell. Dots show parametrizations yielding the behaviors in (**A**) of the corresponding color. The blue ellipse encloses the individual viable space where an individual cost measuring consistency of the single-cell behavior with the design objective for individual cells is below a threshold $\varepsilon$. Red and orange dots encircled by ellipses represent individual cells drawn from the two distinct cell populations. **C** Space of population parameters $\gamma$, where each parameter vector (dot) describes a full distribution of individual parameters in a population, typically via mean vector and covariance matrix. The orange ($\gamma$) and red ($\gamma '$) dots represent the population parameter vectors that generate the corresponding populations in (**A, B**)

**Fig. 2:**
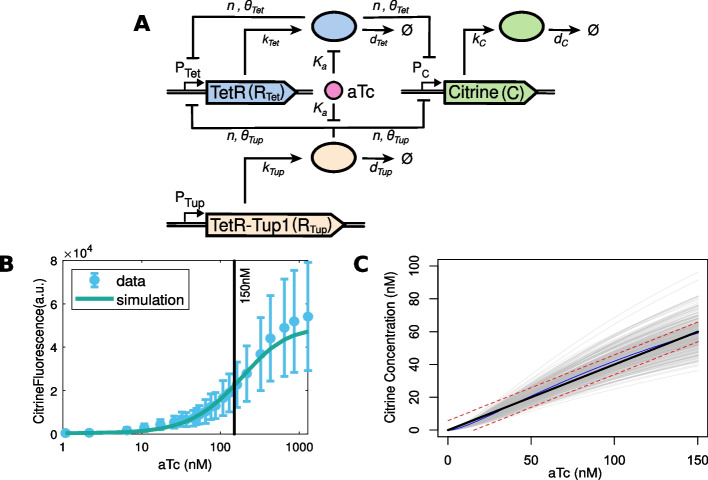
Well-tempered controller (WTC) circuit. **A** Schematic representation of the circuit structure and its parametrization. Rectangles: genes with associated promoters; ellipses: proteins (corresponding color); lines with arrows: molecular reactions; lines with bar heads: regulatory interactions for inactivation. **B** Experimental and simulated aTc dose-response curve for the WTC. Blue: mean (circles) and standard deviation (error bars) of experimental data obtained by flow cytometry; green line: simulation results for the estimated parameter values in Table 1. Additionally, we used estimated values $d_{C} = 0.006 \text { min}^{-1}$, $d_{Tet} = 0.0087 \text { min}^{-1}$, $\theta _{Tet} = 0.006 \text { nM}$, and $\theta _{Tup} = 0.5 \cdot 10^{-4} \text { nM}$. To match the model output (Citrine concentration) to fluorescence (a.u.), we determined a scaling factor as in [4]. **C** Simulated dose-response curves of a population of cells for a given population parameter $\gamma$ with a coefficient of variation $CV \approx 10\%$. Parameter means were the estimated parameters from (**B**). Blue line: mean response; gray lines: responses of individual cells; black line: reference linear dose-response curve; red lines: reference curve $\pm \varepsilon = 6\,\textrm{nM}$; the individual cost threshold; an individual trajectory is viable if it lies within the red curves on average. Note that aTc is in linear scale

Computer-aided design helps identifying suitable network structures (topologies) as well as biological parts for their implementation to reach a given design objective. For the commonly applied models in the form of ordinary differential equations (ODEs), both design problems can be addressed by investigating the space of model parameters to assess (predicted) circuit behaviors in relation to design objectives encoded by a reference for the desired behavior. With sampling-based methods such as (approximate) Bayesian computation, this defines a ‘viable’ subspace of the parameter space where the behavior is consistent with the design objective (Fig. 1A, B) [3–5].

The ODE-based approach captures the behavior of an ‘average’ cell and thus only allows design with respect to such an assumed cell. Yet, for the biological implementation it is critical that a circuit functions under conditions of uncertainty (e.g., in changing environmental conditions or because the models do not capture relevant interactions between parts or with the cellular context [6]) as well as cell-to-cell variability that is present even in isogenic populations (e.g., due to extrinsic or intrinsic stochastic noise, or different cell cycle phases and ages of cells in a population [7]). One can account for uncertainty in ODE-based design, for example, via measures of robustness that quantify parameter uncertainty [4]. It is also possible to tackle cell-to-cell variability with stochastic models, where temporal logic specifications are written as Continuous Stochastic Logic (CSL) [8]. However, the pure ODE and CSL frameworks are limited in two main aspects: First, they cannot account for all aspects of cell-to-cell variability directly; stochastic models do not represent extrinsic variability resulting, for example, from variable cell sizes. This is particularly important when an ‘average’ cell poorly represents the population dynamics, for example, when subpopulations of cells show different qualitative behaviors. Second, and related, it is not possible to define design objectives for the population, such as requiring a certain fraction of the cells to have a coherent behavior.

To address these limitations, here we propose a framework for robust synthetic circuit design that takes into account cell-to-cell variability, and clearly separates it from experimental noise and impact of variable environmental conditions and interacting parts. For this population design, we extend MCMC based sampling approaches for ODE-based design [4] to the NLME (Non-linear Mixed-Effects) models framework [9]. Specifically, this entails augmenting the ODE model with a statistical model at the population level that induces probability distributions over the parameter space at the individual cell level (see Fig. 1B, C). This allows a designer to impose cell-to-cell variability constraints on synthetic networks. Additionally, we propose an efficient MCMC algorithm with parallel tempering (PT) [10] to scale our approach to larger population design problems. We demonstrate the approach with the a posteriori analysis of a recently developed transcriptional controller [11], a class of circuits that is often designed to minimize cell-to-cell variability.

## Results

### Population design framework

For any individual cell, we assume that the dynamics of the synthetic circuit are governed by the *individual cell model*

1 $$\begin{aligned} \Sigma (\beta ): {\left\{ \begin{array}{ll} \frac{dx(t)}{dt} = v(x(t), u(t), \alpha ) \\ x(0) = x^0 \\ y(t) = h(x(t))\;, \end{array}\right. } \end{aligned}$$

where *x* are the system states such as concentrations of chemical species, *v* is a rate function, and *u* is an input function. Usually, states cannot be observed directly and the observations *y* of the system result from a (known) observation function *h*. We subsume the parameters $\alpha$ and initial conditions $x^0$ into the parameter vector $\beta =(\alpha , x^0)\in B$, where *B* is a bounded set.

We first consider the *individual cell design problem* of determining the parameter $\beta ^*$ that minimizes the divergence between the circuit’s behavior and a desired reference behavior. We model the *behavior of* $\Sigma$ as an input–output map $D:\mathbb {R}\times B\times \mathcal {U}\rightarrow \mathbb {R}$ that provides a (time-dependent) function $D(\tau ; \beta ,u)$ in $\tau$ for each parameter vector $\beta \in B$ and any input $u\in \mathcal {U}$, where $\mathcal {U}$ is a finite set of relevant inputs. The *reference behavior* $D^\text {ref}:\mathbb {R}\times \mathcal {U}\rightarrow \mathbb {R}$ is a user-specified (time-dependent) function for each $u\in \mathcal {U}$ that encodes the desired input–output relation; it need not be realizable by $\Sigma$. A simple example is a dose-response curve, where a constant input *u* is mapped to a constant response for the reference, and to the output at steady state for $t\rightarrow \infty$ for the circuit. Another example identifies $D(\tau ; \beta ,u)=y(\tau )$ as the observations of $\Sigma$ at time $\tau$ for a given input and parameter.

We measure the divergence between system and reference behavior by the *individual cost function*

2 $$\begin{aligned} s(\beta )=\frac{1}{|\mathcal {U}|}\sum _{u\in \mathcal {U}} \left| \left| D(\tau ; \beta ,u)- D^\text {ref}(\tau ; u)\right| \right| \;, \end{aligned}$$

which averages some norm $||\cdot ||$ between the system and reference behavior over the considered inputs.

In principle, the individual cell design problem could be solved directly to identify the optimal individual cell parameter $\beta ^*=\text {argmin}_\beta s(\beta )$. However, additional uncertainties arise due to unmodelled system components and from combining previously characterized biological parts into a circuit [12]. We account for these uncertainties by defining a threshold $\varepsilon >0$ on the cost function to encode which solutions are ‘good enough’, and determine the *viable region* $V^\text {ind}=\{\beta \in B\mid s(\beta )\le \varepsilon \}$ of all parameters that fulfill this criterion. An output of the individual cell design problem is then a description of $V^\text {ind}$ rather than a single parameter.

To capture cell-to-cell variability, we postulate a *population model*, where all cells share the same model structure $\Sigma$, but each cell *i* has its own parameter $\beta _i$ drawn from a common *population distribution*

3 $$\begin{aligned} \beta _i \sim P_\gamma \end{aligned}$$

with *population parameters* $\gamma \in \Gamma$. This is known as a *nonlinear mixed-effects model* and $P_\gamma$ is often chosen to be a normal or log-normal distribution, in which case $\gamma$ are the expected values and (co)variances of the parameters in $\beta _i$. We assume that the distribution $P_{\gamma }$ admits a probability density function (p.d.f.) $p_{\gamma }(\beta )$ for all $\gamma \in \Gamma$.

The population model allows us to consider the distribution of behaviors of a circuit under cell-to-cell heterogeneity. In particular, each population parameter $\gamma$ yields a specific distribution $P_\gamma$ of the individual cell parameters $\beta$, and this induces a distribution over the values of the individual cost functions $s(\beta )$. The *population design problem* then consists of finding a population parameter that minimizes a corresponding *population cost function*, given by a functional

4 $$\begin{aligned} c:\{P_\gamma \mid \gamma \in \Gamma \} \rightarrow \mathbb {R}^+\;. \end{aligned}$$

For example, $c(\gamma )=\mathbb {E}_\gamma (s(\beta ))$ considers the expected value of the individual costs over the population, and $c(\gamma )=\mathbb {P}_{P_\gamma }(s(\beta )\ge \varepsilon )=\mathbb {P}_{P_\gamma }(\beta \not \in V^\text {ind})$ considers the percentage of cells whose behavior deviates from the reference by more than a user-defined threshold $\varepsilon$ (cf. Fig. 1B); this percentage depends on the specific population distribution $P_\gamma$, and therefore on the population parameter $\gamma$.

Again, the population design problem can in principle be solved directly to yield $\gamma ^*=\text {argmin}_\gamma c(\gamma )$. Here, we again relax this problem and seek to identify the *population viable space* $V^\text {pop}=\{\gamma \in \Gamma \mid c(\gamma )\le \delta \}$ to account for additional uncertainties, where $\delta$ is again a user-defined parameter. In particular for design objectives such as requiring a minimal fraction of cells with ‘acceptable’ behavior that will have multiple optima, the population viable space also yields equivalent design alternatives.

To sample the viable spaces, we previously applied a naive MCMC-based algorithm to a low-dimensional design problem, but it proved to be slow and had a poor mixing [13]. It would be increasingly difficult and time-consuming to apply it to larger problems involving more parameters. To overcome these computational limitations for the important case of controlling the percentage of sufficiently well-behaved cells, here we propose and implement an alternative sampling algorithm that we call Stochastic Likelihood Markov Chain Monte-Carlo (SLMCMC). Its general idea is to sample jointly from the population parameter space and the individual parameter space, and then to discard individual parameters (see Methods for details).

### Design problem for a transcriptional controller

To demonstrate the framework, we use a transcriptional controller termed well-tempered controller (WTC) that was experimentally designed by Azizoglu et al. [11]. In the WTC (Fig. 2A), expression of the fluorescent protein Citrine—or of any gene of interest—is regulated by constitutively expressed TetR-Tup1 and by autorepressed TetR. Anhydrotetracycline (aTc) can bind to both TetR and TetR-Tup1, thereby inactivating their ability to repress gene expression.

Experimentally, it was shown that cell-to-cell variability in the expression of Citrine is reduced through the introduction of the TetR-mediated negative feedback. At the same time, the dose-response curve—obtained by adding different amounts of the inducer molecule aTc—was tuned to approach an ideal linear dose-response, corresponding to high Input Dynamic Range (IDR) and high Output Dynamic Range (ODR) [14] (Fig. 2B).

Given that we already know the final network structure of the WTC, we aim to use our computational framework to determine the acceptable characteristics of the distribution of circuit parameters in a population of cells, namely their mean and covariance, such that a large proportion of cells in the population will display a dose-response curve close to an ideal reference curve. Notably, we wish to establish whether our framework can identify the relevance of the feedback mechanism in the context of a population of cells.

**Table 1:** Parameter specifications for the WTC model

Name	Description	Units	Fixed value		Cell-specific
*Fixed parameters*					
$k_{Tet}$	Max production rate of TetR	nM min$^{-1}$	1.12		Yes
$k_{Tup}$	Max production rate of TetR-Tup1	nM min$^{-1}$	0.79		Yes
$k_{C}$	Max production rate of Citrine	nM min$^{-1}$	0.84		Yes
$d_{Tup}$	Degradation constant of TetR-Tup1	min$^{-1}$	1.28		Yes
*n*	Hill coefficient for promoter repression by TetR	(–)	1.57		No
$K_a$	Association constant for TetR and TetR-Tup1 binding to aTc	nM$^{-1}$	144.37		No
$U^{nM}_{sc}$	Scaling constant: nM per tdh3 producing unit (see [4])	nM $\cdot$ unit$^{-1}$	0.76		No

Name	Description	Units	Bounds	Explored in log space	Cell-specific
*Sampled parameters*					
$d_{Tet}$	Degradation constant of TetR	min$^{-1}$	[$3 \cdot 10^{-3}$ 0.1]	Yes	Yes
$d_{C}$	Degradation constant of Citrine	min$^{-1}$	[$3 \cdot 10^{-3}$ 0.1]	Yes	Yes
$\theta _{Tet}$	Repression coefficient TetR	nM	[$10^{-3}$ $10^{6}$]	Yes	No
$\theta _{Tup}$	Repression coefficient TetR-Tup1	nM	[$10^{-6}$ 20]	Yes	No

Parameters $k_{Tet}, k_{Tup}, k_C, \text {and } d_{Tup}$ are cell-to-cell variable but their means are fixed to the indicated values. *S. cerevisiae*’s typical growth rate in YPD medium is $0.0077\,\textrm{min}^{-1}$ [4]. To account for dilution and potential cell-to-cell variability, lower bounds for all degradation constants were fixed to $3e-3\,\textrm{min}^{-1}$

**Table 2:** Sampling efficiency in the population parameter space

Criterion	MCMC	SLMCMC				
Samples	20,000	200,000				
Covariance	Scalar	Scalar				Diagonal
*κ*	–	3	{3,5}	{3,7}	{3,9}	{3,7}
Run-time (s)	23,755	900	2639	3624	3713	3413
Checking-time (s)	0	615	721	719	725	746
Rejected samples (%)	0	4	1.33	1.33	0.5	2.33
minESS	157	1505	3011	3345	2981	1487
Time/sample (s)	1.188	0.0076	0.017	0.020	0.022	0.021
Time/minESS (s)	151.31	1.01	1.12	1.19	1.49	2.80
Sampling efficiency (%)	0.79	0.75	1.51	1.67	1.49	0.74

Runs were performed on a standard laptop with Intel i7 processor for the population sampling problem with $\varepsilon = 6\textrm{nM}$. Run-time is the sampling time. Checking-time is the time to naively compute population costs for 600 sampled populations (unnecessary for the naive approach). Populations with cost above the threshold account for the rejected samples. minESS is the minimum of the individual ESSs obtained across multiple dimensions. Sampling efficiency is the ratio between minESS and the actual number of samples drawn. Ratios involving time are computed by summing over run-time and checking-time

We first formulated an ODE model to describe the behavior of the WTC circuit (see Fig. 2A) for an individual (‘average’) cell. The model involves the concentration of the input molecule aTc (*a*)—which can be added to the cell culture—and three states for the total concentrations of the repressor TetR, the repressor TetR-Tup1, and the fluorescent protein Citrine (see Methods for details). We estimated the model’s 10 parameters using data from Azizoglu et al. [11] using least-squares non-linear optimization (see Table 1). Note that compared to our earlier study [13], we make the model biologically more realistic by accounting for dilution due to cell growth through the degradation constants. As shown in Fig. 2B, the parametrized WTC model captures the experimental dose-response curve well. In particular, the model can give a response close to linear in the input range shown in Fig. 2C. Here, the parameterized model serves only to generate a realistic reference behavior; therefore we did not investigate model aspects such as identifiability.

To define the design problem, we encode this observed behavior as the reference behavior. Specifically, our objective for the behavior of individual cells endowed with the WTC is a linear dose-response curve over an IDR of $[0\,\textrm{nM}, 150\,\textrm{nM}]$ for aTc with a desired ODR of $[0\,\textrm{nM}, 60\,\textrm{nM}]$. We define our individual cost $s(\beta )$ (Eq. 2) as the $L_2$ distance between an individual cell’s response and the reference dose-response curve. Parameters $\beta$ that fulfill $s(\beta )\le \varepsilon$ constitute the individual viable space, and we consider different values for the threshold $\varepsilon$ (see Methods).

For our population design, we consider the percentage of individual cells in a population with parameter $\gamma$ that fulfill the criterion for acceptable dose-response as our population cost function:

5 $$\begin{aligned} c(\gamma ) = \mathbb {P}_{P_\gamma }(s(\beta ) \ge \varepsilon )\;. \end{aligned}$$

We define the population viable space as those $\gamma$ that yield at least 80% individual cells with behavior sufficiently close to the reference; correspondingly, $c(\gamma )\le 0.2$.

To illustrate the interplay between the individual and the population level in our design problem, Fig. 2C shows an example of the dose-response relationship of the WTC model for a population of cells. The NLME formulation takes into account the variance in parameters, that is, cell-to-cell variability. Here, although the mean response is close enough to the ideal response, many response curves are not within the acceptable range due to variance in the individual parameters. Specifically, we obtain a population cost of $c(\gamma ) \approx 0.3$, given an individual cost threshold of $\varepsilon = 6$ nM that corresponds to approximately 10% deviation from the reference curve. The example illustrates a key difference between traditional design and population design. The traditional design considers the mean response of the population, which is very close to the reference curve, although a significant fraction of individual cells might not comply with the design objective. In contrast, the population design explicitly considers variability and rejects design options if the proportion of non-compliant cells is too high.

### Individual-cell design

We were first interested in identifying the relevance of the feedback mechanism for single-cell responses, focusing on individual costs and (viable) parameter spaces. To simplify the computations as well as the analysis of relationships between parameters, we sampled a four-dimensional parameter space after fixing the means of 6 out of 10 parameters of the ODE model (see Table 1). The four remaining parameters ($d_{Tet}, d_C, \theta _{Tet}$, and $\theta _{Tup}$) are the protein degradation constants, and the effective concentrations relative to the repression (including feedback) mechanisms. This set of parameters also allows to explore limit cases where parts of the network are removed: A high repression constant ($\theta _{Tet}$ or $\theta _{Tup}$) is equivalent to removing the corresponding repressive effect; fixing $d_{Tet}$ or $d_C$ to a large value effectively removes the production of TetR or Citrine.

For sampling, we used a naive, adaptive version of the Metropolis-Hastings algorithm [15] with a pseudo-likelihood based on individual cost (see Methods for details). We selected a threshold of $\varepsilon = 6\,\textrm{nM}$ for the individual cost, corresponding to approximately 10% of the target ODR.

**Fig. 3:**
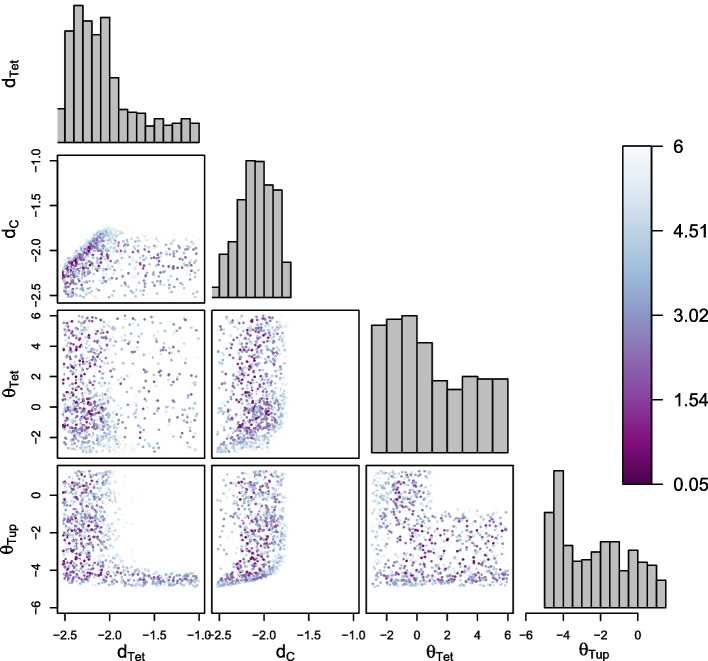
Viable samples in the individual parameter space. Histograms show marginal distributions, and scatter plots samples in all two-dimensional projections of the parameter space. In the projections, samples are colored according to their individual cost from light blue to purple: a darker blue indicates a lower cost, and thus a higher consistency of the WTC dose-response with the reference curve for a given point. Only the parameters present in the plot were allowed to vary, all others were fixed to values specified in Table 1. Additionally, all parameters were sampled in $\hbox {log}_{10}$-scale, and are displayed as such

**Fig. 4:**
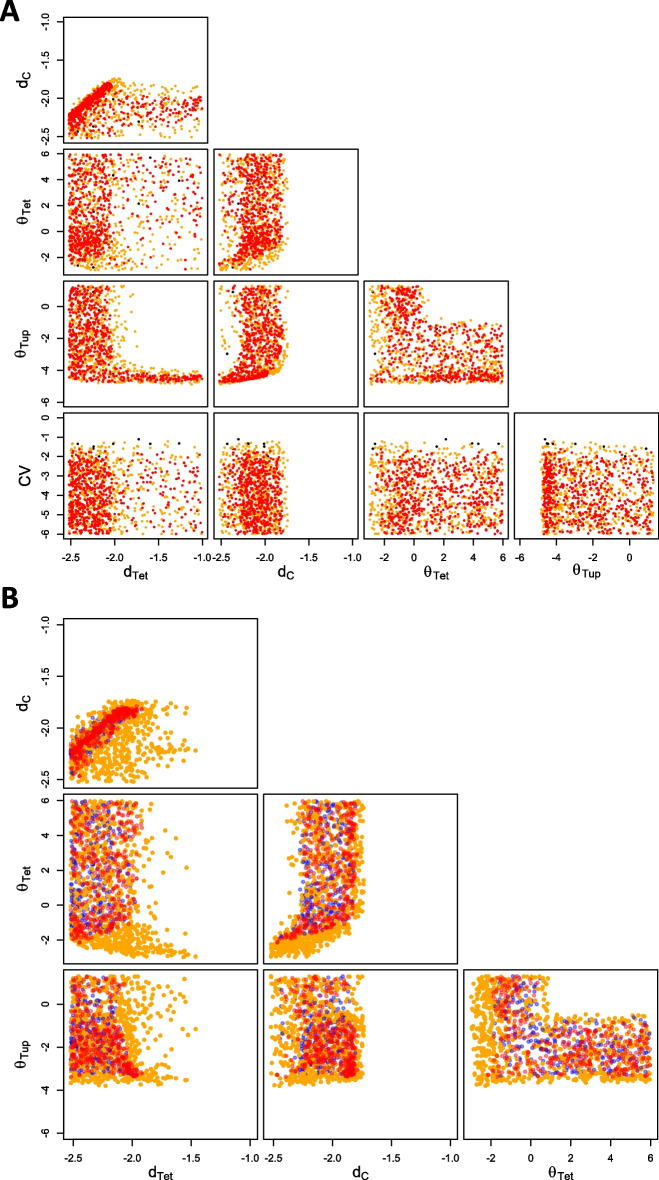
Comparison of individual and population viable spaces. **A** Samples in all two-dimensional projections of the population parameter space obtained with the SLMCMC algorithm for a scalar covariance matrix (less than 1.4% of samples are not viable). *CV* is the common coefficient of variation for all cell-to-cell variable population parameters. Orange (red) dots: viable samples for the threshold on the individual cost $\varepsilon = 6\,\textrm{nM}$ ($\varepsilon = 3\,\textrm{nM}$); black dots: populations with cost $c(\gamma ) > 0.2$. All parameters are in $\hbox {log}_{10}$-scale. **B** WTC model with increased cooperativity of repression. Orange (blue) dots: Individual samples with cost above (below) $\varepsilon = 3\,\textrm{nM}$, individual threshold $\varepsilon = 6\,\textrm{nM}$. Red dots: Population samples with fixed $CV = 7\%$, population threshold $\delta = 0.2$

In Fig. 3, we first note that the protein degradation constant of Citrine, $d_C$, displays a substantially narrower marginal distribution than all other parameters. Citrine is the system response, and therefore this distribution shape is not surprising: with all other parameters kept identical, a change in $d_C$ will directly impact the shape of the dose-response curve.

The two-dimensional projections of the joint distribution over the individual viable space $V^\text {ind}$ exhibit a correlation between the two parameters for protein degradation, $d_{Tet}$ and $d_C$, mainly in the region of low individual cost $s(\beta )$. This indicates that either of the two degradation constants could be used to fine-tune the circuit.

The two parameters $\theta _{Tet}$ and $\theta _{Tup}$ capture the strengths of auto-repression and constitutive repression, respectively. Higher values correspond to weaker repression, but do not exclude other vital roles for TetR (resp. TetR-Tup1) in the circuit. Values for the full range of $\theta _{Tet}$ and $\theta _{Tup}$ are viable individually. However, the two parameters cannot take high values simultaneously, indicating that effective repression of at least one type is needed for the circuit to achieve the desired behavior.

A high degradation constant $d_{Tet}$ might allow us to remove TetR from the circuit design and rely on constitutive repression alone. However, looking at the $(d_{Tet}, \theta _{Tup})$ projection reveals that this option requires precisely controlling constitutive repression by keeping $\theta _{Tup}$ in a very narrow range. This is unlikely to be feasible in a biological implementation—keeping TetR in the circuit therefore seems advisable even without a repressive role.

For low values of $\theta _{Tet}$ and $\theta _{Tup}$, these two parameters are also correlated with the degradation constant of Citrine, $d_C$, because stronger repression needs to be compensated by slower degradation to allow mean expression of Citrine in the desired range. Citrine mean expression seems less affected by lower repression, where this correlation vanishes.

Considering viable parameter values jointly thus provides insights into parameter restrictions and correlations. It also identifies robustness with respect to parameter perturbations that can be exploited for circuit design.

### Population design

We next applied the complete population design framework to the WTC model to obtain design guidelines for a reasonably good transcriptional controller with low cell-to-cell variability in the steady-state dose-response. Specifically, we set a population threshold of $\delta =0.2$, requiring at least 80% of the cells in a population to meet the individual design goal.

Table 1 summarizes the parameter specifications. Assuming log-normal distributions, we sample only variances ($k_{Tet}$, $k_{Tup}$, $k_C$, and $d_{Tup}$), only means ($\theta _{Tet}$ and $\theta _{Tup}$), or both ($d_{Tet}$ and $d_C$). While variances are unlikely to be tunable in practice, sampling cell-to-cell variability of parameters allows us to identify maximum admissible values compatible with the design objectives and hence to select suitable biological parts (of known and fixed variance) for the circuit.

As a main extension to our prior work [13], we here introduce the SLMCMC algorithm for sampling in population design. The previous, naive approach is computationally inefficient because substantial sampling of individual parameters is required to impose the hard threshold $\delta$ on the population cost in Eq. 6. We reasoned that we might gain efficiency by relaxing the hard constraint during sampling, focusing on sampling viable population parameters with sufficient probability, and checking the hard $\delta$-constraint *a posteriori*. For details on the algorithm, see Methods.

To test the algorithm, we first assume an identical coefficient of variation $CV = \sqrt{e^{\sigma ^2}-1}$ for all parameters that are cell-to-cell variable. The covariance matrix of the underlying Normal distribution is then $\sigma ^2\cdot I$. Since $\sigma \le 0.1$ in our viable samples, we used the approximation $CV \approx \sigma$ to simplify our analysis slightly.

**Fig. 5:**
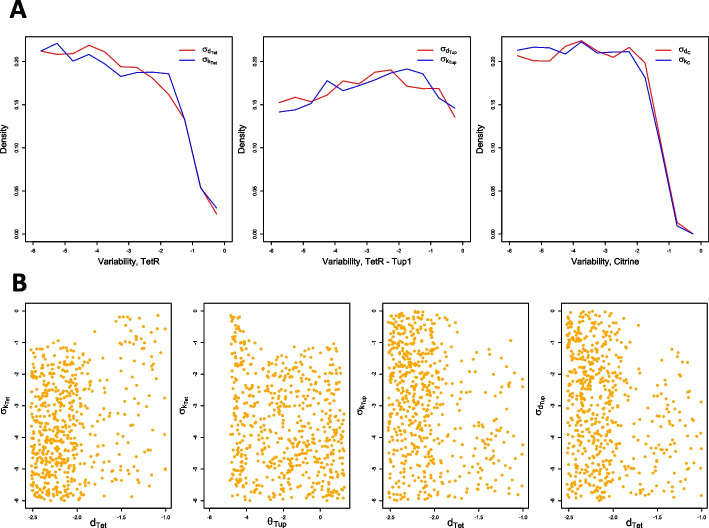
Distributions of sampled population parameters, diagonal covariance matrix. Individual threshold is set to $\varepsilon = 6\,\textrm{nM}$. For the full distributions, see Additional file 3: Fig. S2. **A** Marginal distributions of samples for cell-to-cell variability in the population parameter space. **B** Viable samples in selected two-dimensional projections of the population parameter space

**Fig. 6:**
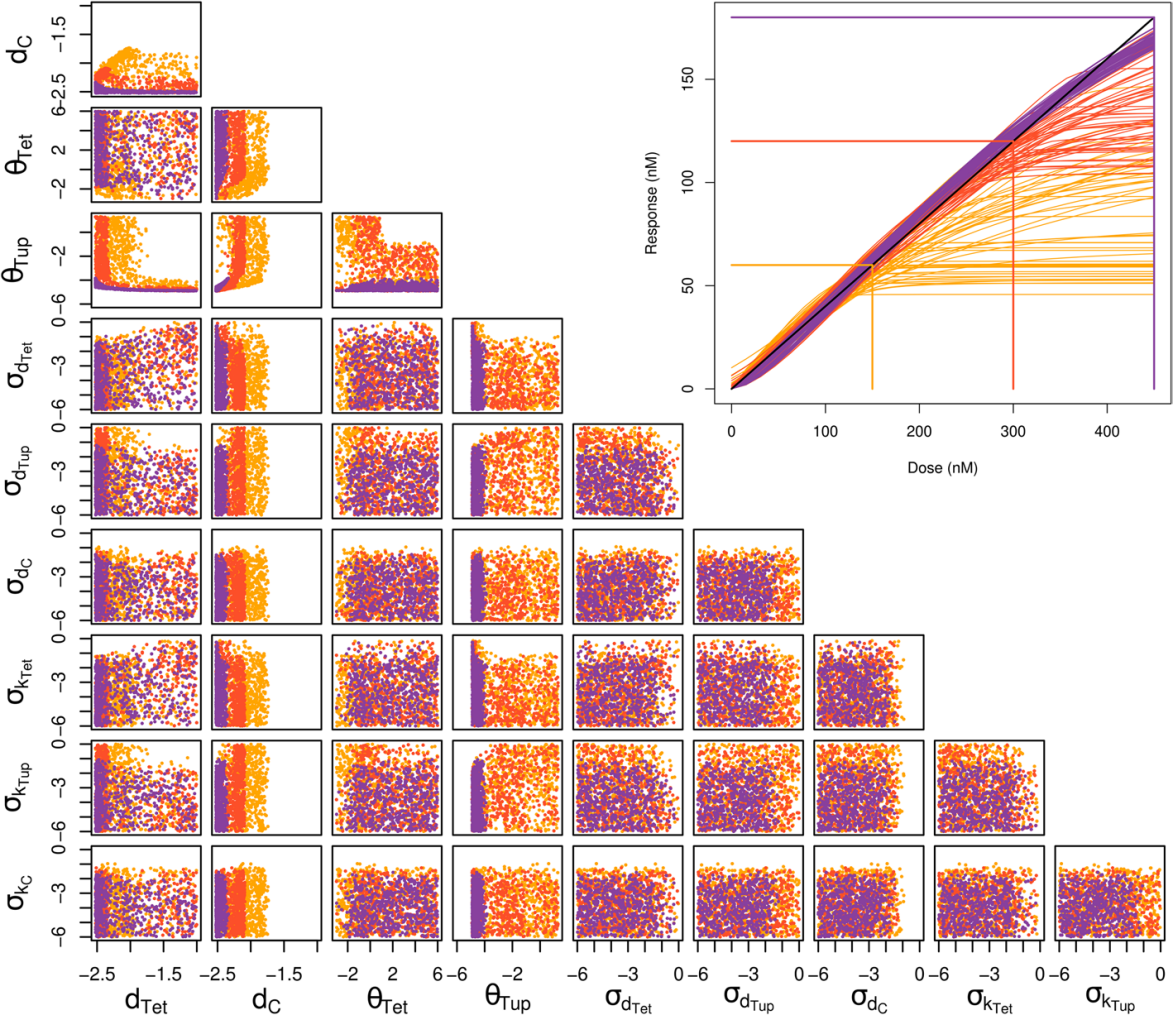
Viable samples in the population parameter space for reference curves with extended IDR. Samples in all two-dimensional projections of the parameter space, obtained with the SLMCMC algorithm for individual threshold $\varepsilon = 6\,\textrm{nM}$ and diagonal covariance matrix. Orange dots: IDR up to $150\,\textrm{nM}$; red dots: IDR up to $300\,\textrm{nM}$; purple dots: IDR up to $450\,\textrm{nM}$ nM. Non-viable population samples (i.e. $c(\gamma ) > 0.2$) were removed prior to plotting. All parameters were sampled in $\hbox {log}_{10}$-scale, and are displayed as such. Top-right corner: Corresponding viable population dose-response curves. Black line: reference curve up to $450\,\textrm{nM}$. Colored lines corresponding to sample colors: 50 population dose-response curves per reference curve, computed as in Fig. 2C from 50 samples of population parameters. Colored boxes delimit the portion of the black line used as a reference curve during population design

Figure 4A shows the resulting population parameter distributions for the SLMCMC algorithm using the two individual cost thresholds $\varepsilon =6\,\textrm{nM}$ and $\varepsilon =3\,\textrm{nM}$. They are very similar to the ones obtained with the naive approach (Additional file 2: Fig. S1), indicating that the SLMCMC approach finds parameters yielding the desired behavior at the population level, although it samples from a different distribution initially (see Methods for details).

For the mean parameters, the patterns are also very similar to those obtained for individual samples (Fig. 3). This is not surprising because we allowed for low values for the variance $\sigma$ in the exploration; for low $\sigma$, essentially all population members behave like the (viable) mean parameters.

The data in Fig. 4A provides new insights, first, because of the two individual thresholds $\varepsilon$ employed. A reduced threshold makes the correlation between $d_{Tet}$ and $d_C$ stronger, highlighting the importance of tuning the two parameters jointly to achieve a stricter objective. In line with the individual design results, the joint distribution of repression parameters $\theta _{Tet}$ and $\theta _{Tup}$ shows that either type of repression needs to be strong to achieve the design objective. In addition, for lower $\varepsilon$, the region of low $\theta _{Tet}$ and high $\theta _{Tup}$ recedes to higher $\theta _{Tet}$, indicating that simultaneous strong auto-repression and weak constitutive repression can be detrimental for stricter objectives.

The second new insight derives from sampling parameter variances in population design. Although we allowed a CV up to one, Viable samples are essentially all below $7\%$ for $\varepsilon = 6$ nM and $2.4\%$ for $\varepsilon = 3$ nM. These values could be maxima for the admissible cell-to-cell variability for reaching the design objective. For future studies, it is, hence, of interest to experimentally quantify the cell-to-cell variability of the parameters, and check the results against our inferred value. Note, however, that higher CVs would be allowed in the presence of negative correlations between parameters.

Here, we sampled the *CV* to identify the maximum admissible variability. To assess the impact of a fixed (high) variability on the viable space, we then fixed $CV = 7\%$. In addition, because non-linearities should make differences between individual and population design more pronounced [16], we increased the Hill coefficient for TetR-Tup1 ($n_{Tup} = 5$). This makes the roles of constitutive repression and auto-repression more distinct. As shown in Fig. 4B, the population viable space becomes restricted to parts of the space where individual cells would have a very low individual cost. As a result, some parameter combinations are no longer viable for populations including variability. For instance, $d_{Tet}$ and $d_C$ have much higher correlation in the population viable space, and will need well-coordinated fine-tuning.

Next, we abandoned the assumption of equal variances and considered a diagonal covariance matrix with diagonal entries $\sigma _{\text {j}}^2$, which increases the parameter space to 10 dimensions. The resulting distribution for the mean parameters is very similar to the one obtained for the scalar covariance matrix (Additional file 3: Fig. S2). However, viable variances differ considerably (Fig. 5A): $\sigma _{d_{Tup}}$ and $\sigma _{k_{Tup}}$ can rise to CVs as high as 1. Thus, TetR-Tup1 degradation and production constants can vary substantially in the population without much impact on the performance. Yet, $\sigma _{d_C}$, $\sigma _{d_{Tet}}$, $\sigma _{k_C}$, and $\sigma _{k_{Tet}}$ are constrained to values below 10%, indicating that narrow distributions for the corresponding constants are critical for adequate circuit performance.

Tight control of $d_C$ and $k_C$ is unsurprising as these parameters are directly related to Citrine, the system’s output. The importance of controlling TetR concentration is less obvious, as $\sigma _{k_{Tet}}$ and $\sigma _{d_{Tet}}$ can rise to higher values for $\varepsilon = 6\textrm{nM}$. However, high values of $\sigma _{k_{Tet}}$ are always associated with high $d_{Tet}$ and a narrow distribution of $\theta _{Tup}$ (Fig. 5B, first two panels). This corresponds to the limiting case of very strong constitutive repression combined with very low amounts of TetR, in which case the variance of TetR is unimportant. Indeed, a high value of $d_{Tet}$ forces a lower variability on TetR-Tup1 parameters $k_{Tup}$ and $d_{Tup}$ (Fig. 5B, last two panels). If TetR is removed from the system (very high $d_{Tet}$ value), TetR-Tup1 must show little cell-to-cell variability and a very precise repression strength.

Considering parameter-specific variances in the population design problem thus provides additional insights because parameters with low variance require tighter control for the circuit to work.

### Sampling efficiency

We quantified the efficiency of the naive approach and of our new SLMCMC approach with and without parallel tempering by recording the run times for generating samples and by computing the minimal Effective Sample Size (minESS) as the minimum Effective Sample Size (ESS) over all dimensions (Table 2).

For the naive approach, we generated 20,000 samples for the population design problem with $\varepsilon =6$ nM. We checked convergence of the chain using the Gelman–Rubin criterion [17], indicating that several thousand samples were needed for convergence. This resulted in a total run-time of more than 23,000 s and an effective sample size of 157. This corresponds to more than 1 s per sample and a sampling efficiency of 0.79%. Importantly, though, the naive approach ensures that all sampled population parameters fulfill the population design criterion of at least 80% of the cells meeting the individual target.

In contrast, our SLMCMC approach does not guarantee the hard population threshold; it requires post-processing of the sampled parameters to check if the target was indeed met. We distinguish these two stages in our calculations. The SLMCMC does not require estimating the population cost in each step—a very costly computation. This allows generating samples much more efficiently, and we therefore generated 200,000 samples. Using a parameter $\kappa =3$, a scalar covariance matrix, and no parallel tempering, these samples are generated in about 900 s. We argue that it is not necessary to check the population design criterion for each sample, and use a statistical approach instead: we check the criterion only for a randomly picked subsample of 600 out of the 200,000 samples. This provides a reliable estimate of the percentage of population parameters that do not meet the hard threshold. We found that only an estimated 4% of the samples did not meet the population design criterion. The minimal ESS is comparable to the naive approach, as is the sampling efficiency.

To evaluate the impact of parallel tempering on performance, we chose chains with $\kappa _1=3$ and $\kappa _2=5,\,7,\,9$. The run-time for generating 200,000 samples increased to about 2.8–4-fold compared to the non-tempered approach, but the percentage of samples not meeting the target dropped to 0.5–1.3%. The minimal ESS roughly doubled, leading to an overall sampling efficiency of about 1.5%, double the efficiency of the naive and the non-tempered approaches. We again selected 600 samples at random to check the population design criterion, which added about 700 s to the overall computation time.

Finally, we selected the most efficient parallel tempering setting with $\kappa _1=3$ and $\kappa _2=7$ and used a diagonal instead of a scalar covariance matrix. The overall run-time is comparable to the previous settings, but the fraction or rejected samples increased slightly to about 2.3%. More importantly, several variance parameters need to be sampled in this setting, and the increased problem dimension leads to a minimum ESS of 1487, about half of the other PT-based scenarios. The sampling efficiency is then comparable to the naive approach and the non-tempered SLMCMC for scalar covariance matrices.

Overall, our SLMCMC algorithm with parallel tempering outperforms the naive approach independent of the specific setting for the inverse temperatures. It generates about two orders of magnitude more effective samples per time, shows an order of magnitude increase in minimal ESS and a massive reduction in overall run-time.

### Extended population design

Finally, we asked if population design could help to extend the WTC’s input dynamic range. Specifically, starting from the current input dynamic range with maximum $\textrm{maxIDR}=150\,\textrm{nM}$, we aimed to extend it to $300\,\textrm{nM}$, $450\,\textrm{nM}$, and $600\,\textrm{nM}$, respectively, while restricting the slope of the dose-response curve to the current value. We used the SLMCMC approach with an individual threshold of $\varepsilon = 6\,\textrm{nM}$ and a diagonal covariance matrix to sample the population parameter space. The top-right of Fig. 6 presents examples of populations for each of the three design objectives, alongside the corresponding reference curves.

Most population parameters are little affected by extending the IDR, but the maximal viable values for $d_C$ and $\theta _{Tup}$ decrease considerably with increasing $\textrm{maxIDR}$ (Fig. 6). The viable space for $\textrm{maxIDR}=450\,\textrm{nM}$ is restricted to very low values of these two parameters, which limits options for implementation severely. However, auto-repression seems less important in this case, and $\theta _{Tet}$ becomes less restricted.

For variance parameters, patterns are similar to those observed previously, with the exception that parameters related to TetR-Tup1 become more constrained with increasing $\textrm{maxIDR}$. For high $\textrm{maxIDR}$, auto-repression does not help to achieve the design objective and the variances of $k_{Tup}$ and $d_{Tup}$ become independent of $d_{Tet}$. Meanwhile, high variances of $d_{Tet}$, $k_{Tet}$ remain viable only for high $d_{Tet}$. This indicates that if TetR is present in the system (low $d_{Tet}$), its variability should be controlled, as its binding with aTc may otherwise propagate variability to the fraction of bound TetR-Tup1 and further to Citrine.

The observed parameter restrictions become untenable for $\textrm{maxIDR}=600\,\textrm{nM}$ and our method found no viable population parameter set for this case. Indeed, with a minimum individual cost above $8\,\textrm{nM}$, no individual cell can satisfy the individual cost requirement given the WTC model’s parametrization and constraints on the parameter spaces.

Thus, our population design approach suggest that–and how–the WTC’s input dynamic range can be extended, and it gives an indication of the limits to such an extension under the current circuit design (and parametrization).

## Discussion

Nearly all current methods for synthetic circuit design assume an ‘average’ cell that needs to be optimized to fulfill the design objectives, potentially by considering parameter variations to achieve robustness of the biological implementation [4]. Stochastic design frameworks that account for cell-to-cell variability due to intrinsic noise with low molecule copy numbers are beginning to emerge, but computational complexity currently limits them to small networks, steady-state, and homogeneous model parameters in a cell population [18]. Here, we therefore proposed population design via NLMEs as an alternative to both approaches. We argue that it has the potential to bring information about cell-to-cell variability to synthetic biological design in realistic settings, and to help infer the impact of said variability on the system of interest. We additionally propose the SLMCMC algorithm as an efficient way to solve the population design problem *via* sampling.

Our case study considers a problem synthetic circuit designers often face, namely to tune their system in order to reduce cell-to-cell variability [11].

Repression mechanisms were necessary both in the individual and population cases. This indicates that constitutive repression and auto-repression are useful to linearize dose-response curves of individual cells. Interestingly, TetR seemed to be able to fulfill that role even when it does not enact auto-repression.

The population sampling with scalar covariance matrix highlighted $7\%$ as a possible maximum admissible coefficient of variation, whereas our analysis with a diagonal covariance matrix showed that the variances of different parameters play different roles. For the WTC, parameters related to Citrine and TetR have to have low cell-to-cell variability, but TetR-Tup1 permits higher variability, as long as TetR is present in the system.

Constitutive repression increased the admissible *CV* from $\approx 4\%$ to $\approx 7\%$. However, we do not know if constitutive repression had a direct impact on cell-to-cell variability, or if it simply helped linearize the dose-response curve. To disentangle these possibilities, we would need to define a measure of variability reduction independent of the shape of the response, and to compare this measure for different repression strengths. Additionally, any variability reduction will be directly linked to repression strength: increasing repression would decrease cell-to-cell variability as well as mean expression of the repressed component. To weaken or eliminate this link between mean and variability, one may need to consider more complex circuit topologies [19, 20].

In view of enhancing the WTC’s performance, a more restrictive design objective (larger IDR and ODR) could be obtained by tuning precisely the constitutive repression strength and the degradation constant of Citrine. Constitutive repression proved essential to increase IDR and ODR, in contrast to auto-repression. At least with the given model and parametrization, however, we do not predict that the highest IDR we tested can be achieved by the WTC.

Note that some of the analysis results for the WTC differ from our previously published results [13]. There, we used the same model, but lower bounds on degradation constants for a proof-of-concept. Additionally, we fixed the maximal simulated time to 1000 min, which is a reasonable time in an experimental setting. Here, we accounted for effective degradation constants closer to the experimentally observed dilution rate, leading to increased lower bounds and a different design objective. In addition, we adjusted simulation times for the system to reach steady-states. These changes in combination explain the differences between the present and the earlier study.

In terms of methods, our new sampling technique for population parameters, the SLMCMC algorithm, proved reliable and fast as it bypasses the need to compute the population cost. The added benefit of the parallel tempering approach is an increased mixing, especially when the target distribution is multimodal with poorly connected regions of high density. SLMCMC worked efficiently for our rather high-dimensional applications, but it is still an MCMC algorithm, and as such it becomes less efficient in higher dimensions. To reduce this limitation, SLMCMC could be combined with techniques such as evolutionary MCMC [21] to improve mixing. However, because the number of variance parameters (including correlations) grows quadratically with the number of individual parameters, it is likely that one will not be able to tune the variance of each parameter individually for large models. Instead, one could fix the covariance matrix to estimates obtained from experimental data, for example, by using well-established NLME inference approaches [7, 9]. A complementary approach would be to approximate the individual cost [22]. Other alternatives, which are not compatible with SLMCMC, include small sets sampling techniques such as the sigma-point approximation [23], or replacing exact MCMC sampling by approximate methods. For example, variational inference can be much faster than MCMC and still give accurate results, provided that the correlation structure of the likelihood is properly accounted for [24].

Here, we explored the population parameter space of a network topology we knew should work for some parameter values. In the broader context of synthetic biology, a working, simple topology that has the potential to achieve the design objective is not necessarily known. In many cases, one may want to explore different topologies and select the one that performs best while still being simple enough. To achieve this goal while taking into account cell-to-cell variability, we propose to apply a method similar to that described by Lormeau et al. [4] to the objective function defined at the population level. Briefly, the algorithm will explore a number of possible topologies by simplifying an initial (complex) starting network. The viability (existence of parameters making the network viable) of each network is assessed. One can then choose robust networks according to the size of the viable region, for instance.

Overall, the population design framework could then be used to recommend network structures, together with their parameter values, that are best suited to fulfill a design objective incorporating cell-to-cell variability. Such an approach could also help exploring situations where cell-to-cell variability and a given distribution over behaviors of cells in a population is desirable. One example is bet-hedging in bacterial populations, where non-genetic variability across a population increases the chances of survival in the face of antibiotics [25].

## Conclusions

Our general framework for population design, along with the efficient SLMCMC algorithm, aim to help biologists interested in synthetic circuit design to account for cell-to-cell variability via ODE-based NLMEs. The a posteriori demonstration of its usefulness for a transcriptional controller shows scaling to a relevant problem size, although the implementation suffers from classic sampling inefficiency. In perspective, an extension to topology design could enable the rational design of synthetic gene circuits that induce prescribed (distributions of) behaviors at the population level, and thereby allow to exploit cell-to-cell variability for novel applications.

## Methods

### Overview

Our main objective is a description of the population viable space $V^{\text {pop}}$ that encompasses all solutions of the population design problem. As this space will typically not have an explicit description, we resort to MCMC methods to sample it. We propose three sampling strategies: a naive MCMC approach, a new method that exploits joint sampling of the individual and population parameter spaces which we call *Stochastic Likelihood MCMC (SLMCMC)*, and a modified version of SLMCMC that uses parallel tempering to increase sampling efficiency. We restrict attention to our WTC problem and use the proportion of viable individual cells as our population design criterion. The naive MCMC approach readily generalizes to other population objectives, but the SLMCMC approach is currently tailored to this specific objective.

### Naive algorithm for population sampling

Our previously described naive sampling approach [13] is a straightforward implementation of the Metropolis-Hastings sampler. We directly use the population design criterion as our pseudo-likelihood:

6 $$\begin{aligned} L(\gamma ) = \mathbbm {1}\left( c(\gamma ) \le \delta \right) \, . \end{aligned}$$

The resulting samples from the population parameter space $\Gamma$ will have a p.d.f. proportional to $L(\gamma )$, which constrains them to the population viable space. The function $L(\gamma )$ is not amenable to direct calculation because the population cost $c(\gamma )$ is not known explicitly. We therefore draw *N* random individual parameters $\beta _i$ from the corresponding population distribution $P_\gamma$ to approximate $c(\gamma )$ by $\hat{c}(\gamma )=\sum _{j=1}^{N} \mathbbm {1}(s(\beta _j) \le \varepsilon )/N$ and $L(\gamma )$ by $\hat{L}(\gamma )=\mathbbm {1}\left( \hat{c}(\gamma ) \le \delta \right)$. We use $N=300$ for our computations, which provided sufficient sample size for our problem. The full approach is given in Algorithm 1, where we use *c* and *L* instead of $\hat{c}$ and $\hat{L}$ for readability.
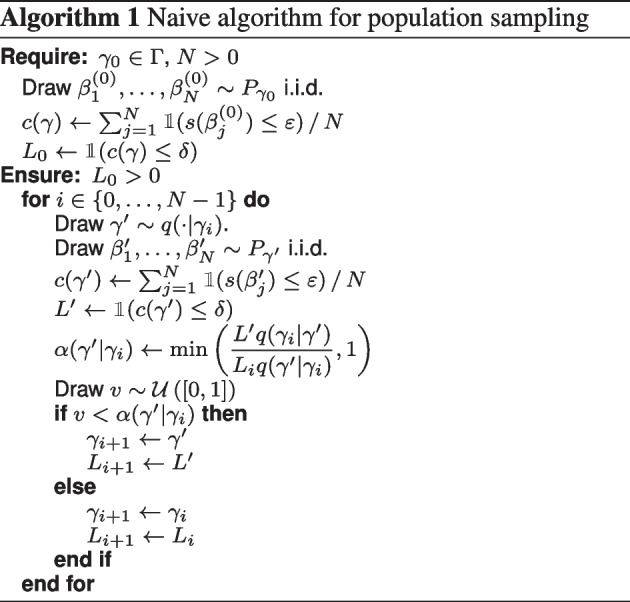


### SLMCMC algorithm

To derive the algorithm, note that Eq. 6 can be rewritten as

7 $$\begin{aligned} L(\gamma ) = \mathbbm {1}\left( \mathbb {P}_{P_\gamma }(s(\beta ) > \varepsilon ) \le \delta \right) \, , \end{aligned}$$

where $\beta \sim P_\gamma$ is considered a random variable. Removing the indicator and the population threshold, and taking the complement probability yields the objective

8 $$\begin{aligned} L(\gamma ) = \mathbb {P}_{P_\gamma }(s(\beta ) \le \varepsilon ) = 1-c(\gamma ) \end{aligned}$$

which provides the desired relaxed version of Eq. 6.

The central idea is then to extend this objective to the product space $\Gamma \times B$ of population and individual parameters:

9 $$\begin{aligned} L(\gamma , \beta ) = \mathbbm {1}\left( s(\beta ) \le \varepsilon \right) \cdot p_\gamma (\beta )\;, \end{aligned}$$

where $p_\gamma$ is the density of the log-normal distribution $P_\gamma$. Evaluating Eq. 9 and sampling from the joint distribution is straightforward. The relaxed objective Eq. 8 is then the marginal

10 $$\begin{aligned} L(\gamma ) = \int _B L(\gamma ,\beta ) d\beta \;, \end{aligned}$$

MCMC sampling from $L(\gamma , \beta )$ followed by discarding the $\beta$ component will provide $\gamma$ samples distributed according to the objective (Eq. 8).

In a last step, we introduce a constant exponent $\kappa \in \mathbbm {N}_{>0}$ to concentrate the pseudo-likelihood around favorably high values, a standard technique used in Bayesian design [26], for example. This yields the objective

11 $$\begin{aligned} L(\gamma ) = \mathbb {P}_{P_\gamma }^\kappa (s(\beta ) \le \varepsilon )\;, \end{aligned}$$

which we sample using the corresponding joint pseudo-likelihood

12 $$\begin{aligned} L(\gamma , \beta _1, \dots , \beta _\kappa ) = \prod _{j=1}^\kappa \mathbbm {1}(s(\beta _j) \le \varepsilon )\cdot p_\gamma (\beta _j)\;. \end{aligned}$$

Intuitively, this means that instead of a single random individual parameter yielding sufficiently low individual cost, we now require that $\kappa$ independent parameters simultaneously yield low costs. The higher $\kappa$ is, the lower the population costs of the sampled parameters will be on average and the more stringent the following sampling will be.

We propose Algorithm 2 to sample from the joint distribution (Eq. 12). We use a Metropolis-Hastings algorithm with the custom proposal distribution:

13 $$\begin{aligned} q(\gamma ', \beta _1', \ldots , \beta _{\kappa }' | \gamma , \beta _1, \ldots , \beta _{\kappa }) = q(\gamma ' | \gamma ) \prod _{j = 1}^{\kappa } p_{\gamma '}(\beta _j') \, , \end{aligned}$$

where $q(\gamma ' | \gamma )$ is a user-defined proposal distribution, restricted to the population parameter. In other words, we first draw a proposal for the population parameter; this proposal depends on the last population parameter, but not on the last individual parameters. After that, we draw the proposal for the individual parameters directly as independent samples from $P_{\gamma '}$. This cancels the conditional probability terms in the acceptance ratio. We thus need to store $\prod _{j = 1}^{\kappa } \mathbbm {1}\left( s(\beta _j) \le \varepsilon \right)$, but not the individual parameters.
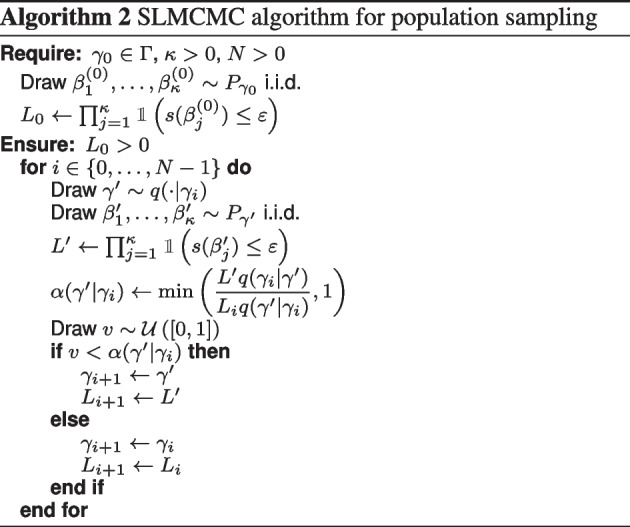


Two technical aspects are worth noting: first, $L_0$ and $L'$ are products of indicator functions; we can stop sampling values $\beta _i$ as soon as the first indicator function is zero to further reduce the computational burden. Second, sampled individual parameters $\beta _i$ are already ‘likely’ as they are drawn from the population distribution with parameter $\gamma$ and therefore follow the required conditional distribution. Hence, we do not need to compute the conditional probabilities directly. This would also allow us to replace $P_\gamma$ with more complex distributions that do not admit a p.d.f. in closed form, but from which it is reasonably easy to sample.

An important difference between standard Metropolis-Hastings and our variant is the computation of the likelihood ratio, which is stochastic in our algorithm (hence the name SLMCMC). An equivalent technique is known as likelihood-free sampling or subset simulation in the context of Bayesian models, where it is applied to sampling datasets rather than parameters [27, 28].

### SLMCMC with parallel tempering

Our SLMCMC approach for sampling the viable population space is still an MCMC technique and suffers from the associated drawbacks, particularly poorly scaling with problem dimension and poor mixing for multimodal distributions. Poor scaling can be addressed using adaptive proposals [15, 29, 30], and we now describe a parallel tempering (PT) [31] extension to improve mixing.

Parallel tempering uses several parallel chains with limiting distributions forming a sequence from a ‘flat’ to the target distribution. A popular family of distributions uses an inverse temperature parameter $\lambda \in [0,1]$ and limiting distributions of the form $f^\lambda$, where *f* is the desired target. Chains with higher temperature (lower $\lambda$) have ‘flatter’ distributions which makes it easier to explore the sampling space and avoid local modes. In addition, two chains with limiting distributions *f* and $f'$ can ‘swap’ their current states *x* and $x'$ with probability

14 $$\begin{aligned} \alpha _{\text {swap}}(f, f') = \frac{f\left( x'\right) f'\left( x\right) }{f\left( x\right) f'\left( x'\right) } \,. \end{aligned}$$

Swapping states renders the individual chains non-Markovian, but the set of chains remains Markovian on the product space with the product distribution of the chains as its invariant distribution [10].

To apply the parallel tempering approach to SLMCMC, we use the number of individual parameters $\kappa$ to provide the inverse temperature. Specifically, consider the pseudo-likelihood Eq. 11 with exponent $\kappa _{\max }$ as our sampling target. We specify a sequence of *r* chains whose targets are the pseudo-likelihoods Eq. 12 with different values $\kappa _1<\kappa _2<\cdots <\kappa _r=\kappa _{\max }$ for the parameter $\kappa$. This generates a family of *r* distributions for parallel tempering with inverse temperatures $\kappa _i/\kappa _{\max }$.

These chains have different state-spaces $\Gamma \times B^{\kappa _i}$, which prohibits direct application of the swapping procedure. We therefore extend the pseudo-likelihoods to the highest-dimensional state-space $\Gamma \times B^{\kappa _{\max }}$ and write the likelihood for the $\kappa _i$ chain as

15 $$\begin{aligned} L_{\kappa _i}(\gamma , \beta _1, \ldots , \beta _{\kappa _{max}}) = \prod _{j = 1}^{\kappa _{max}} \mathbbm {1}\left( s(\beta _j) \le \varepsilon \right) ^{\mathbbm {1}(j \le \kappa _i)} p_{\gamma }(\beta _j) \;. \end{aligned}$$

This effectively sets the ’acceptance’ of parameters $\beta _j$ to one for $j>\kappa _i$. With this provision, and applying Eq. 14, the acceptance probability to swap between two chains with parameters $\kappa <\kappa '$ and samples $\beta _i, \beta _i'$, respectively, is

$$\begin{aligned} \alpha _{\text {swap}}(\kappa , \kappa ')&= \min \left( \frac{ \prod _{j=1}^{\kappa } \mathbbm {1}(s(\beta _j') \le \varepsilon ) \prod _{j=1}^{\kappa '} \mathbbm {1}(s(\beta _j) \le \varepsilon )}{\prod _{j=1}^{\kappa } \mathbbm {1}(s(\beta _j) \le \varepsilon ) \prod _{j=1}^{\kappa '} \mathbbm {1}(s(\beta _j') \le \varepsilon )} , 1\right) \\&= \min \left( \prod _{j = \kappa +1}^{\kappa '} \frac{\mathbbm {1}(s(\beta _j) \le \varepsilon )}{\mathbbm {1}(s(\beta _j') \le \varepsilon )} , 1\right) \\&= \prod _{j = \kappa +1}^{\kappa '} \mathbbm {1}(s(\beta _j) \le \varepsilon ) \; \end{aligned}$$

because $\mathbbm {1}(s(\beta _j') \le \varepsilon ) = 1$ for $j>\kappa$ and the product cannot exceed one in any case.

Our SLMCMC approach with parallel tempering is given in Algorithm 3. Note that the required numbers of samples $\beta _j$ depend on the tempering parameters $\kappa _i$: for two chains with $\kappa _1<\kappa _2$, we need $\kappa _1+\kappa _2$ samples of individual parameters to update the two chains, and additional $\kappa _2-\kappa _1$ individual parameters for swapping, that is, $2\cdot \kappa _2$ sampled individual parameters in total.
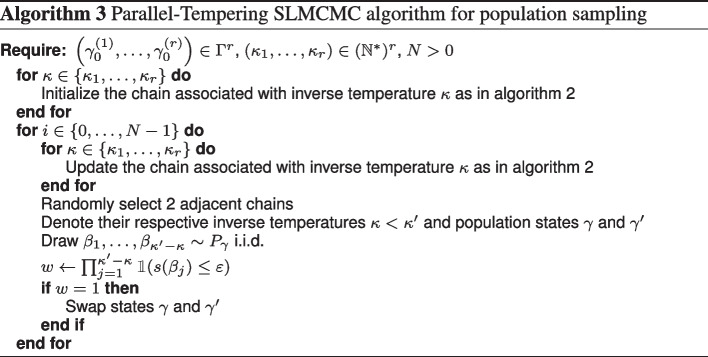


### WTC model

We model the dynamics of the total concentrations of the repressor TetR ($R_{Tet}$), the repressor TetR-Tup1 ($R_{Tup}$) and the fluorescent protein Citrine (*C*) by:

16 $$\begin{aligned} \frac{\textrm{d} R_{Tet}}{\textrm{d} t}&= \frac{k_{Tet}}{ 1 + \left( \frac{f \cdot R_{Tet} }{\theta _{Tet}}\right) ^n + \left( \frac{f \cdot R_{Tup}}{\theta _{Tup}}\right) ^n } - d_{Tet} \cdot R_{Tet} \end{aligned}$$

17 $$\begin{aligned} \frac{\textrm{d} R_{Tup}}{\textrm{d} t}&= k_{Tup} - d_{Tup} \cdot R_{Tup} \end{aligned}$$

18 $$\begin{aligned} \frac{\textrm{d} C}{\textrm{d} t}&= \frac{ k_C}{ 1 + \left( \frac{f \cdot R_{Tet} }{\theta _{Tet}}\right) ^n + \left( \frac{f \cdot R_{Tup}}{\theta _{Tup}}\right) ^n } - d_{C} \cdot C . \end{aligned}$$

Parameters $k_{Tet}$, $k_{Tup}$ and $k_{C}$ are maximal expression constants that capture both transcription and translation to keep the model simple. Parameters $d_{Tet}$, $d_{Tup}$ and $d_{C}$ are the degradation constants.

The two Hill functions represent control terms for TetR and Citrine production, respectively. They depend on the active concentrations of the repressors TetR and TetR-Tup1. Active TetR and TetR-Tup1 molecules are those that are not bound to the inducer aTc. Assuming rapid equilibrium for the binding of aTc to TetR and TetR-Tup1 (as in Lormeau et al. [4]), the fraction of active TetR and TetR-Tup1 (*f*) is given by:

19 $$\begin{aligned} f&= \frac{1}{2} - \frac{1 + K_a a - \sqrt{(1 + K_a(R_{Tet}+R_{Tup}-a))^2 + 4 K_a a}}{2 K_a (R_{Tet} + R_{Tup})} . \end{aligned}$$

Experimental data showed that TetR and TetR-Tup1 have different repression efficiencies [11], represented by $\theta$ in the model. We therefore decided to model the action of the two repressors on their controlled genes as an ‘OR’-gate. This means that we are not taking into account that the repressors might bind to the same DNA sequences. In contrast, we do not expect a difference in Hill coefficient (*n*) or affinity ($K_a$) to aTc between TetR and TetR-Tup1. The SBML version of the model is available as Additional file 1.

We used the ‘deSolve’ package [32] to solve the ODE model. The final time for simulations was set to $25\times 10^6$ minutes. To ensure that the model reached steady state, we increased simulation time when the relative variation in Citrine observed over the last 500 min exceeded $10^{-10}$.

### WTC design problem

The steady-state dose-response curve as a reference behavior takes the aTc concentration *a* as a constant input $u(t)\equiv a$, and yields a constant response $D^\text {ref}(a)\equiv D^\text {ref}(\tau ; a)$ for all $\tau$. We encode the high-IDR, high-ODR objective by defining $(a, D^\text {ref}(a))$ to be the straight line between $(0\,\textrm{nM},0\,\textrm{nM})$ and $(150\, \textrm{nM}, 60\, \textrm{nM})$.

To quantify the deviation between an individual cell’s behavior and the reference curve, we use the individual cost from Eq. 2 based on the dose-response curve $(a, D(\tau ; \beta , a))$, where cell *i* has individual parameter set $\beta _i=($ $k_{Tet}^{(i)}$, $k_{Tup}^{(i)}$, $k_C^{(i)}$, $d_{Tup}^{(i)}$, *n*, $K_a$, $d_{Tet}^{(i)}$, $d_C^{(i)}$, $\theta _{Tet}$, $\theta _{Tup})$, and $D(\tau ; \beta , a)\equiv D(\beta , a)$ is the steady-state ($t \rightarrow \infty )$ response to aTc concentration *a*. In our implementation, the individual cost function is calculated via a discrete version of the $L_2$-norm based on *N* aTc input doses $\mathcal {U}=\{a_1, \dots , a_N\}$, regularly spaced (every $25\,\textrm{nM}$) between 0 and $150\,\textrm{nM}$:

20 $$\begin{aligned} s(\beta ) = \sqrt{\frac{1}{N}\sum _{k = 1}^N \left( D(\beta ,a_k) - D^\text {ref}(a_k) \right) ^2}\;. \end{aligned}$$

### Sampling parameter spaces for the WTC

We defined the pseudo-likelihood for the individual parameter space as:

21 $$\begin{aligned} l(\beta ) = \mathbbm {1}(s(\beta ) \le \varepsilon ) \end{aligned}$$

with $\varepsilon \in \{6\,\textrm{nM}, 3\,\textrm{nM}\}$, therefore sampling uniformly the viable region $V^\text {ind}=\{\beta \in B\mid s(\beta )\le \varepsilon \}$.

For the naive sampling approach, the pseudo-likelihood for the population parameter space was:

22 $$\begin{aligned} L(\gamma ) = \mathbbm {1}(c(\gamma ) \le \delta ) \end{aligned}$$

with $\delta = 0.2$. We then obtain uniformly distributed samples from the population viable space $V^\text {pop}=\{\gamma \in \Gamma \mid c(\gamma )\le \delta \}$. Note that, as $c(\gamma )$ depends on the value of $\varepsilon$ (Eq. 5), $L(\gamma )$ and the associated population viable space will also depend on its value.

To compute the population pseudo-likelihood (Eq. 22), however, we need to approximate $c(\gamma )$, as it is the functional of a distribution (here, a probability). For each value of $\gamma$, 300 individual parameters were drawn randomly from the underlying log-normal distribution $P_\gamma$. For each individual parameter vector, we computed the individual cost *s* and approximated $c(\gamma )$ as the fraction of samples with individual costs above the corresponding threshold $\varepsilon \in \{6\,\textrm{nM}, 3\,\textrm{nM} \}$.

Note that we are interested in the resulting distribution of the individual costs and not in describing $P_\gamma$. Thus, although we consider six cell-to-cell variable parameters, a sample size of $N=300$ individual parameters proved sufficient to reliably represent this distribution of individual costs as the underlying distance measure between a constant reference and the output of an ODE model is sufficiently smooth. An illustration is given in Fig. 2C, where 300 individual dose-response curves from a population distribution with high coefficient of variation cover the graph sufficiently.

The log-normal population distribution for our example allows us to reduce the required amount of random sampling and to provide more consistent results for the approximation of the population pseudo-likelihood. Note that we can reconstruct the mean vector $\mu \in \mathbb {R}^6$ and the $6\times 6$ covariance matrix *C* of the underlying multivariate Normal distribution from the population parameter $\gamma$. We therefore once generated 300 samples $S_i$ from the standard multivariate Normal distribution *N*(0, *I*) in $\mathbb {R}^6$. For each value of $\gamma$, we constructed the corresponding samples of the individual parameters as $\beta _i=\mu +C^{1/2}\cdot S_i$, where $C^{1/2}$ is the lower triangular matrix from a Cholesky decomposition of *C*. This ensures that repeated calls to our approximation of the population cost function with the same population parameter $\gamma$ yields the same cost and requires only a single sample of size $N=300$.

For SLMCMC sampling, the pseudo-likelihood for the population parameter space was:

23 $$\begin{aligned} L(\gamma ) = P_{\gamma }^{\kappa }(s(\beta ) \le \varepsilon ) \end{aligned}$$

with $\kappa > 1$ and, again, $\varepsilon \in \{6\,\textrm{nM}, 3\,\textrm{nM}\}$. This form of the population pseudo-likelihood will result in a higher density of samples in regions of space that have a lower population cost. This is different from the behavior of the uniform population pseudo-likelihood used for naive sampling, which does not discriminate between two populations as long as both their population costs are below the threshold $\delta$.

For the scalar covariance matrix, we ran parallel tempering with inverse temperatures $\{3\}, \{3, 5\}, \{3, 7\}, \{3, 9\}$, therefore requiring a (maximum) total of respectively 3, 10, 14 and 18 individual samples per step, accounting for the swapping step. In the last three cases, the inverse temperature 3 was present to improve mixing only, and we kept only the samples associated with the higher inverse temperature. We sampled 200,000 populations, removed the first half as burn-in, and then took 600 populations by regularly thinning the samples. An approximation of $c(\gamma )$ was computed using the same approach as for naive sampling. Non-viable populations (i.e., with $c(\gamma ) > 0.2$) were discarded, and we compared the fractions of rejected populations for the different $\kappa$ values. For the diagonal covariance matrix problem, we fixed $\kappa = 7$ because this choice yielded most samples with population cost distributed between 0 and $\delta$, and a small fraction (always smaller than 6.5%) higher than $\delta$. This was assessed *a posteriori* on obtained samples as well.

### Sampling efficiency

To compute the Effective Sample Size (ESS) for each dimension, we used the R package ‘coda’ [33]. The numbers shown in Table 2 were computed only once on a standard laptop, and are subject to variation. Additionally, we expect the numbers involving ESS to be highly problem-dependent, even on the same machine using the same software.

### Supplementary information

**Additional file 1.** SBML model for the WTC This SBML file describes the model used to estimate the WTC’s parameters. The C file used to simulate the model during sampling is provided in the Gitlab repository https://gitlab.com/csb.ethz/slmcmc_applications.

**Additional file 2: Figure S1**. Viable samples in the population parameter space, scalar covariance matrix, obtained with the naive method. Symbols as Fig. 4A.

**Additional file 3: Figure S2**. Viable samples in the population parameter space, diagonal covariance matrix. Samples in all two-dimensional projections of the parameter space, obtained with the SLMCMC algorithm. Orange dots: viable samples for the threshold on the individual cost $\varepsilon = 6\,\textrm{nM}$; red dots: viable samples for $\varepsilon = 3\,\textrm{nM}$; black dots: populations with cost $c(\gamma ) > 0.2$, representing less than 4.5% of samples. All parameters are in $\hbox {log}_{10}$-scale.

## Data Availability

The slmcmc package is available at https://gitlab.com/csb.ethz/slmcmc. The code used to generate and analyze samples for the WTC case study are available in the slmcmc_applications repository, https://gitlab.com/csb.ethz/slmcmc_applications.

## Abbreviations

ODE: Ordinary differential equation
CSL: Continuous stochastic logic
NLME: Non-linear mixed-effects
p.d.f.: Probability density function
MCMC: Markov chain Monte-Carlo
SLMCMC: Stochastic likelihood Markov chain Monte-Carlo
WTC: Well-tempered controller
aTc: AnhydroTetracycline
IDR: Input dynamic range
ODR: Output dynamic range
CV: Coefficient of variation
ESS: Effective sample size
MinESS: Minimal effective sample size
PT: Parallel tempering
