# Supplementary figures and images for: Efficient design of synthetic gene circuits under cell-to-cell variability
Source: BMC Bioinformatics. 2023 Dec 7;24(Suppl 1):460. doi: 10.1186/s12859-023-05538-z (PMC10701960; doi:10.1186/s12859-023-05538-z)

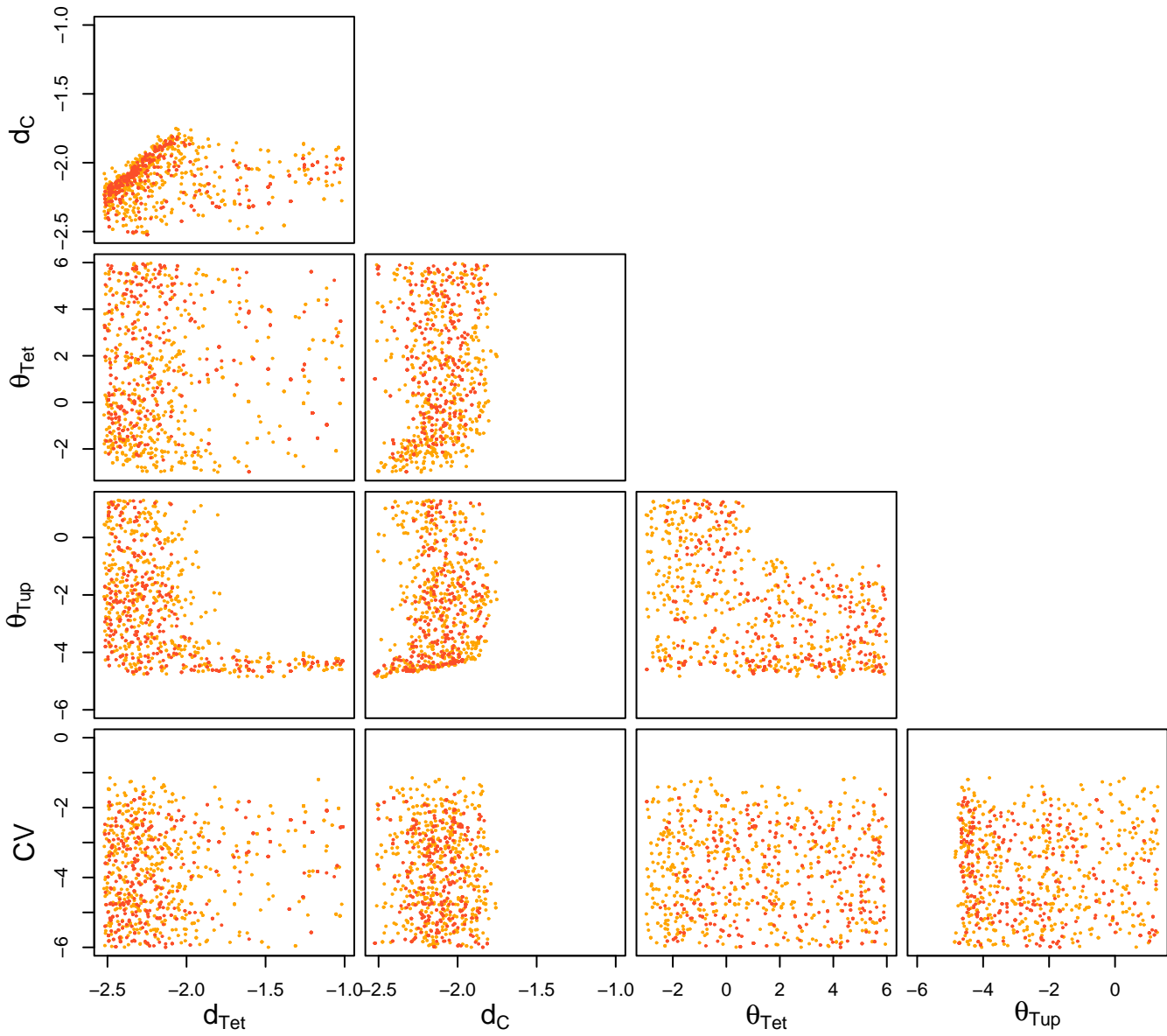

Supplement: Supplementary file 2 — Additional file 2: Figure S1. Viable samples in the population parameter space, scalar covariance matrix, obtained with the naive method. Symbols as Fig. 4A. [file 12859_2023_5538_MOESM2_ESM.pdf]

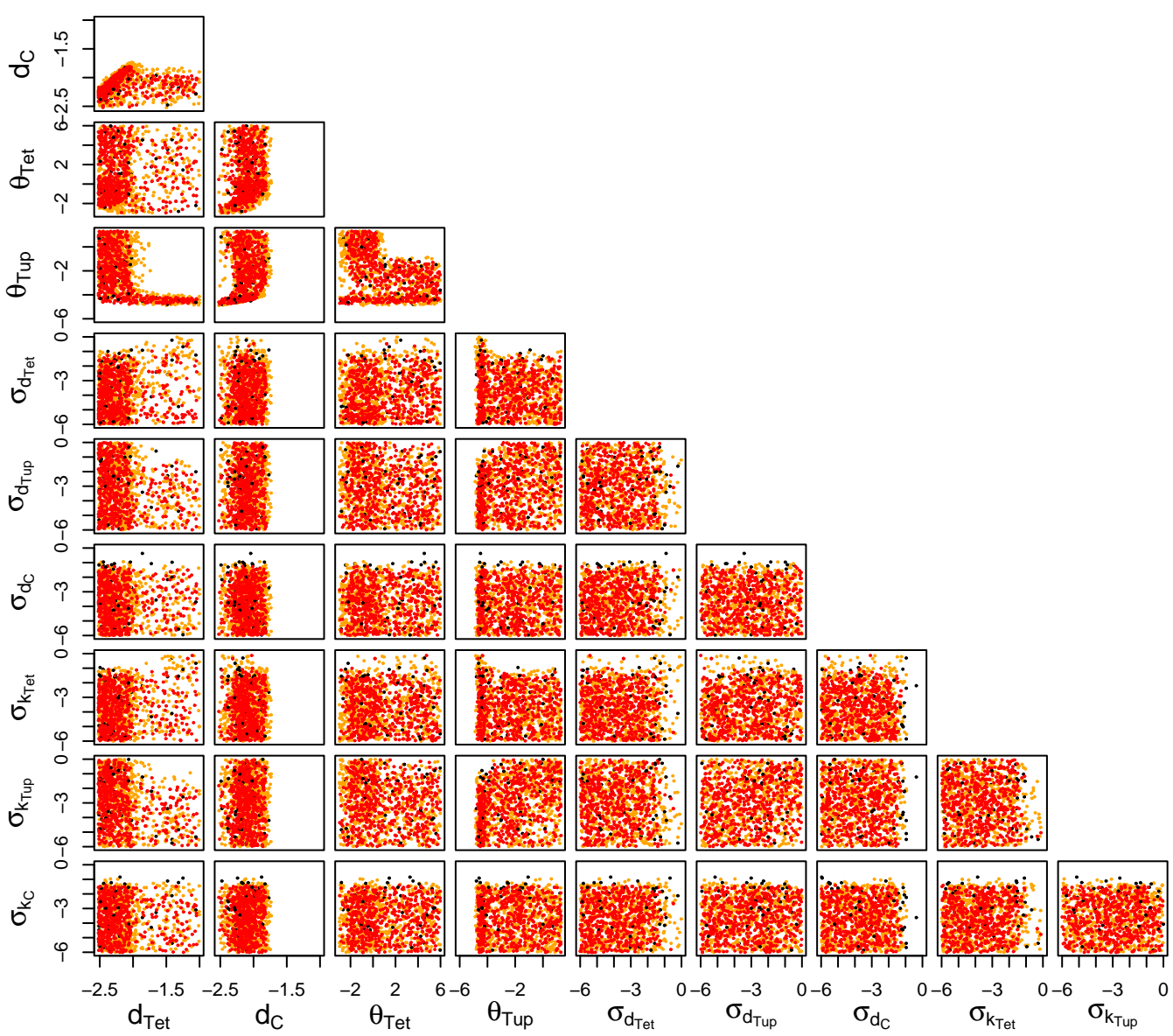

Supplement: Supplementary file 3 — Additional file 3: Figure S2. Viable samples in the population parameter space, diagonal covariance matrix. Samples in all two-dimensional projections of the parameter space, obtained with the SLMCMC algorithm. Orange dots: viable samples for the threshold on the individual cost $\varepsilon = 6\,\textrm{nM}$ε=6nM; red dots: viable samples for $\varepsilon = 3\,\textrm{nM}$ε=3nM; black dots: populations with cost $c(\gamma ) > 0.2$c(γ)>0.2, representing less than 4.5% of samples. All parameters are in $\hbox {log}_{10}$log10-scale. [file 12859_2023_5538_MOESM3_ESM.pdf]
